# Intranasal zinc and vitamin A treatments alter response to bovine respiratory syncytial virus and *Mannheimia haemolytica* co-infection

**DOI:** 10.1093/tas/txaf115

**Published:** 2025-08-25

**Authors:** Emma L Rients, Carlos E Franco, Stephanie L Hansen, Jodi L McGill

**Affiliations:** Department of Animal Science, Iowa State University, Ames, IA, USA; Department of Veterinary Microbiology and Preventive Medicine, Iowa State University, Ames, IA, USA; Current Address: Royal Veterinary College, University of London, London, United Kingdom; Department of Animal Science, Iowa State University, Ames, IA, USA; Department of Veterinary Microbiology and Preventive Medicine, Iowa State University, Ames, IA, USA

**Keywords:** Cattle, bovine respiratory disease, intranasal, vitamin A, zinc

## Abstract

During disease, there may be increased local demands for zinc (Zn) and vitamin A to support pathogen response. This study evaluates the effects of intranasal Zn and vitamin A treatments on steers experimentally infected with bovine respiratory disease (BRD) pathogens, bovine respiratory syncytial virus (BRSV) and *Mannheimia haemolytica*, hypothesizing that steers treated with Zn and vitamin A (VA) will have improved recovery to BRD challenge. Forty-eight Angus crossbred steers (333 ± 4.2 kg) were utilized in two groups with identical challenge timelines. The day prior to challenge (d −1), steers were shipped for 6 hours. On d 0, steers were administered an aerosol inoculation with ~10^4^ TCID_50_/mL BRSV strain 375 followed by an intratracheal inoculation with *M. haemolytica* (1.42 × 10^8^ CFU strain D153, serotype A1) on d 5. On d 4, steers received intranasal treatments: zinc (IN ZN; 50 mg Zn oxide nanoparticles), vitamin A (IN VA; 200,000 IU as retinyl palmitate), a combination of zinc (50 mg) and vitamin A (200,000 IU; IN VA + ZN) or no treatment (CON). Statistics were analyzed using the Mixed procedure of SAS 9.4 (Cary, NC) and contrast statements were utilized to determine the effects of Zn, VA and intranasal treatment. Disease challenge resulted in mostly mild, subclinical signs of disease. There was an interaction for plasma VA (TRT × Day *P* < 0.01) where VA treated steers (IN VA and IN VA + ZN) had sustained plasma VA concentrations on d 5, when ZN and CON had decreased plasma VA. After challenge (d 19), liver VA concentrations were increased in IN VA (IN VA *P* = 0.03) and IN ZN (IN ZN *P* = 0.05) treated steers. Zn treated steers (ZN and ZN + VA) tended to have increased gene expression of *matrix metalloproteinase 9* (*P* = 0.06) on d 5 and *cellular retinol binding protein 1* (*P* = 0.08) on d 7 in cells collected from nasopharyngeal swabs. Additionally, immune cell populations from bronchoalveolar lavage were altered with increased CD11b expression on neutrophils (IN VA *P *= 0.01) and CD11c on macrophages (IN ZN *P* = 0.08) on d 7. During a mild disease challenge, intranasal Zn and VA treatments impacted lung inflammatory environment and nutritional immunity, suggesting potential benefits in mild or deficient nutritional statuses.

## Teaser

The response to disease alters micronutrient homeostasis in the body, despite their importance in the response to pathogens. This research investigates intranasal zinc and vitamin A treatments to a subclinical bovine respiratory disease challenge, proving beneficial for maintaining circulating vitamin A concentrations and the lung inflammatory environment.

## INTRODUCTION

Vitamin A has many key roles in the body, including immune defenses. With roles in mucus production (Manna et al., 1994) and T cell responses (Iwata et al., 2003), vitamin A is crucial for respiratory pathogen defense. Zinc (Zn) also plays many key roles throughout the body and immune system. Zinc deficiency has often been associated with decreased immune response to respiratory disease in humans (Sadeghsoltani et al., 2022) and supplementation has been associated with fewer respiratory infections in humans (Roth et al., 2010). Zinc also supports the immune response through epithelial barrier function in an ex vivo human model (Roscioli et al., 2017), development and function of immune cells in mice and in vitro models (Rolles et al., 2018; Kido et al., 2019) and antiviral properties in vitro (te Velthuis et al., 2010).

Zinc and vitamin A are essential for the metabolism of each other. Zinc is essential in the absorption and transport of vitamin A as a part of retinol binding protein (Satre et al., 2001). First discovered in rats, Zn is also required for the conversion of vitamin A precursors to the active form retinoic acid through the enzyme retinol dehydrogenase (Huber and Gershoff, 1975). In addition, vitamin A deficiency in chicks has resulted in reduction of some Zn binding proteins involved in the absorption of dietary Zn (Berzin and Bauman, 1987). Micronutrient metabolism is relatively conserved across species; therefore Zn and vitamin A have these functions in cattle, despite being discovered in other models.

Vitamin A and Zn are critical for mounting a robust immune response to pathogens. Although dietary supplementation of Zn and vitamin A may be beneficial during a disease challenge, cattle generally decrease feed intake during disease (Chirase et al., 1991; Broadway et al., 2021). Additionally, inflammation can cause the sequestration of Zn (Aydemir et al., 2012) and vitamin A (Rosales et al., 1996), decreasing circulating concentrations. By administering these nutrients at the site of infection, such as intranasally, cells that are responding to pathogens have direct access to the nutrients that may help encourage a more robust immune response. Previously, intranasal vitamin A has supported the immune response to viral infection in vitamin A deficient mice (Surman et al., 2014). Additionally, a meta-analysis investigating the use of Zn as a mitigation strategy for respiratory infections concluded that Zn may decrease symptoms and shorten the duration of respiratory tract infections (Hunter et al., 2021). Administering vitamin A and/or Zn after the onset of disease symptoms could be a tool to reduce the severity and length of infection in cattle.

The current study aims to investigate the effects of intranasal Zn and vitamin A treatments when given to respiratory disease challenged beef steers. The hypothesis was that steers treated with Zn and vitamin A would have improved recovery from disease challenge.

## METHODS

All protocols were approved by the institutional animal care and use committee (IACUC-21-003) and institutional biosafety committee (IBC-21-001).

### Animals and Housing

Sixty Angus crossbred steers, purchased from a single ranch, arrived at the Iowa State University Beef Nutrition farm (BNF) approximately 1 mo prior to challenge and were handled frequently to ensure quiet temperament for this intensive study. Prior to purchase, blood samples were collected from 10 random animals for analysis of bovine respiratory syncytial virus (BRSV) neutralizing titers (160 ± 27.4) to determine if the cohort would be candidates for this study. Samples were submitted to the Iowa State Veterinary Diagnostic lab.

Forty-eight steers (333 ± 4.2 kg) were chosen from this group to be enrolled in this BRD challenge study. The study was completed in two groups at the Animal Resource Station (ARS) to accommodate pen space and logistics. The disease challenge timeline was identical for each group.

At BNF, steers were housed in pens of 6 head per pen and fed a common diet (Table 1) once daily via wagon mixer into a concrete bunk. While at ARS, cattle were housed in 2 pens (n = 12/pen). Each treatment was equally represented in each pen. While at ARS, the diet was mixed via wagon mixer every 3 to 4 d and delivered to ARS for daily feeding by hand. Daily feed delivery was recorded and any leftover feed was weighed and sampled once cattle returned to BNF. Feed and ort samples were collected for dry matter determination. Average dry matter intakes for the challenge periods were 8.5 ± 0.07 kg and 9.59 ± 0.16 kg for groups 1 and 2, respectively.

**Table 1.** Dietary composition

Ground hay	20
Sweet Bran	50
Dry distiller grains^1^	21.56
Cracked corn	6.5
Limestone	1.5
Salt	0.31
Vitamin A and E premix^2^	0.1
Rumensin 90	0.0135
Trace mineral premix^3^	0.016
Analyzed composition	
DM, %	71.28
Crude protein, %^4^	23.86
NDF, %^4^	33.38
Ether extract, %^4^	5.04
Cu, mg/kg DM	18.7
Fe, mg/kg DM	143
Mn, mg/kg DM	45.9
Zn, mg/kg DM	104

^1^Carrier for micro ingredients.

^2^Premix provided 2200 IU vitamin A and 25 IU vitamin E/kg diet DM.

^3^Provided per kilogram of diet DM: 0.15 mg Co, 10 mg Cu, 20 mg Mn, 0.1 mg Se, 30 mg Zn and 0.5 mg I, from inorganic sources.

^4^Based on TMR analysis from Dairyland Laboratories, Inc., Arcadia, WI.

### Disease Challenge and Sampling Timeline

A timeline of study events can be found in Table 2. Initial liver samples were taken 8 to 10 d before BRSV challenge. Biopsies were collected using methods described by Engle and Spears (2000). Steers were weighed prior to feeding on d −2 and −1. They were trucked for 6 hours on d −1 before arriving at the Iowa State University Animal Resource Station (ARS) in Ames, Iowa. On d 0, steers were inoculated with ~10^4^ TCID_50_/mL BRSV strain 375 via aerosol inoculation as previously described by Sacco et al. (2012). On d 4, steers were administered intranasal treatments (n = 12 steers/treatment) Treatments included: Zn (IN ZN; 50 mg Zn oxide nanoparticles), vitamin A (IN VA; 200,000 IU as retinyl palmitate nanoparticles), a combination of Zn (50 mg) and vitamin A (200,000 IU; IN VA + ZN) or control (CON) receiving no intranasal treatment or sham. On d 5 post viral infection, all steers were administered an intratracheal inoculation with 1.42 × 10^8^ CFU (Group 1 = 2 × 10^8^ CFU; Group 2 = 8.53 × 10^7^ CFU) of *Mannheimia haemolytica* (*MH*) strain D153, serotype A1.

**Table 2.** Disease challenge timeline

d −1	Transit event and arrival to Animal Resource Station
d 0	BRSV^1^ inoculation; BAL^1^, nasal swab and blood collection
d 4	Intranasal treatment administration; blood collection
d 5	Intratracheal *M. haemolytica* inoculation; nasal swab and blood collection
d 7	BAL^1^ and blood collection
d 10	Blood collection
d 14	BAL^1^ and blood collection

^1^BRSV = bovine respiratory syncytial virus; BAL = bronchoalveolar lavage.

Steers remained at ARS and were sampled prior to infections (d 0 and 5) and treatments (d 4) and on d 7, 10 and 13/14. Sampling included blood, nasal fluid, nasal and nasopharyngeal swabs, thoracic ultrasound and rectal temperature. On d 0, 7 and 13/14 bronchioalveolar lavage (BAL) samples were also collected. All sampling occurred in the morning prior to feeding. Rectal temperature was collected using a 30 s digital thermometer (Equate, Bentonville, AR). Group 1 steers remained at ARS for an additional day prior to being trucked back to BNF on d 15. Group 2 was transported back to BNF following sampling on d 13/14 of challenge. Both groups were weighed off truck and returned to pens to rest. Liver biopsies were collected on d 19 and 20 post viral challenge for group 1 and 2, respectively, after return to BNF.

### Challenge Model and Treatments

Bovine respiratory syncytial virus for this study was prepared from a virus stock re-isolated from the lung of an infected animal and passaged less than 3 times on bovine turbinate cells. All steers were inoculated with approximately 5 mL of BRSV inoculum, which took approximately 5 min.

Intranasal treatments were made using copolymers of 1,6- bis-(pcarboxyphenoxy) hexane (CPH) and 1,8-bis(p-carboxyphenoxy)-3,6-dioxaoctane (CPTEG) using methods previously described McGill et al. (2019). Intranasal Zn was purchased as ZnO nanoparticle dispersion (20% by weight in water; Sigma Aldrich, St. Louis, MO) with an average particle size of 40 nm. Intranasal treatments were administered using a syringe with atomizer. Total volume for each treatment was 10 mL, with 5 mL administered in each nostril.

The *MH* was prepared using the same methods as Capik et al. (2015). Briefly, cultures of *MH* were grown on blood agar plates in a non-gassed incubator at 37 °C for 2 d prior to challenge. Isolated colonies were suspended in brain -heart infusion broth and grown at 37 °C for 6 hours. After confirming optical density at 600 nm based on *MH* growth curve, the bacterial in log phase were pelleted (3230 × g for 10 min), washed twice with phosphate buffered saline (PBS) then resuspended in sterile saline. A dose of 60 mL of *MH* followed by 60 mL of sterile saline was used to inoculate the steers. The dose was confirmed by quantitative culture on brain heart infusion agar.

### Clinical Illness Scoring

Calves were monitored once daily for signs of clinical illness by a trained, blinded observer. Calves were scored using an adaptation of the University of Wisconsin Calf Health Respiratory Scoring Chart (McGuirk and Peek, 2014). The scoring chart assigns numbers (0 to 3) based on severity of clinical signs including cough (0 = no cough to 3 = repeated spontaneous coughing), nasal discharge (0 = normal, serous discharge to 3 = copious, bilateral mucopurulent nasal discharge), ocular discharge (0 = normal to 3 = heavy ocular discharge), and ear position (0 = normal to 3 = severe head tilt or bilateral ear drop). For our scoring chart we included an additional category for respiratory effort (0 = no effort to 3 = significant effort).

### Sample Collection and Analysis

Blood was collected via jugular venipuncture into vacutainer tubes and stored until processing at the lab. Plasma (K_2_EDTA) and serum was centrifuged for 20 min at 1,000 × g, aliquoted and stored at −80 °C until analysis. Plasma was utilized for determination of trace mineral (Zn, Cu, Fe) concentration via inductively coupled plasma optical emission spectroscopy (ICP-OES; Optima 7000; PerkinElmer, Waltham, Ma) as described by Pogge and Hansen (2013). Quality control samples (serum UTAK, Valancia, CA; bovine liver from National Institutes of Standards and Technology, Gaithersburg, MD) were included to verify instrument accuracy. Plasma vitamin A concentrations were determined using iCheck Flouro (Bioanalyt, Teltow, Germany) using manufacturer’s instructions. For liver vitamin A, samples were freeze dried and ground finely before utilized for vitamin A analysis using iCheck Flouro.

Nasal swabs were taken and stored in virus isolation media (serum free minimum essential media; MEM). Virus isolations were preformed according to the manufacture’s protocol (MagMAX Viral RNA Isolation kit; Thermo Fisher Scientific, Waltham, MA). Briefly, RNA (400µL) was bound using paramagnetic beads with an RNA binding surface. The beads/RNA were captured on magnets, and contaminants and excess binding solution were washed away. Viral RNA was eluted in an elution buffer. qPCR for the viral NS2 gene was performed using the Taqman RNA to CT 1 step kit (Applied Biosystems, Invitrogen, Waltham, MA) as described in Mahmoud et al. (2020) using a Quant Studio 5 Real-Time PCR machine (Thermo Fisher Scientific). A Ct value less than 39 was used to indicate that animal was positive for viral shedding.

To monitor lung consolidation, thoracic ultrasounds (TUS) were collected and scored using methods described in Porter et al. (2021) with the addition of one extra intercostal space. A total of five locations on the left (ICS 19L, 18L, 14L, 10L, and 9L) and five on the right (ICS 23R, 22R, 18R, 19R, and 14R) were scored for TUS. The IBEX EVO (E.I. Medical Imaging, Loveland CO) was used with the L7HD linear transducer probe (5 to 9 MHz) set to a depth of 11.6 cm (for cattle). Scoring for TUS was done utilizing methods described by Hong et al. (2024).

Bronchoalveolar lavage fluid collection was completed using methods modified from Guerra-Maupome et al. (2019). Briefly, steers were restrained in a chute and halters were used to stabilize and position their head. A sterile silicone, large animal BAL catheter (10 mm x 2.5 mm x 240 cm long, MILA International) was inserted into their nostril and blindly passed into the lung, reaching the bronchus. Sterile saline (180 mL) was introduced to the lower respiratory tract in three aliquots followed by immediate suction to obtain lower airway washes. The BAL fluid was pooled, stored on ice, filtered through sterile filter and centrifuged at 940 × g for 10 min. Cells were washed, counted and aliquoted for flow cytometry, stimulation and qPCR analysis. Cells stored for qPCR were stored in 1 mL of TriZol Reagent (Thermo Fisher Scientific) at −80 °C.

Nasopharyngeal (NP) swabs were collected and stored in serum free MEM on ice until processing. Swabs were vortexed prior to removing the swab from the media and centrifuging at 2000 rpm for 10 min. The media was removed from the cells pelleted at the bottom of the tube and the cells were stored for qPCR in 1 mL of TriZol reagent at −80 °C.

Isolation of RNA from BAL and NP cells was completed using MagMAX microarrays total RNA isolation kit (Thremo Fisher Scientific), according to the manufacturer’s instructions. The concentration of RNA was determined using a Qubit RNA broad range kit (Thermo Fisher Scientific) and Quibit 4 fluorometer (Thermo Fisher Scientific). The RNA was DNase- treated and cDNA was synthetized using random primers and Superscript III Reverse transcriptase following the manufacturer’s instructions (Invitrogen, Life Technologies). For BAL, 1000 ng of RNA was used and for NP, 500 ng of RNA was used.

Power SYBR green PCR master mix (Applied Biosystems, Waltham, MA) was used for qPCR analysis on a QuantStudio 5 Real-Time PCR machine (Thermo Fisher Scientific). Expression for each gene was normalized to the housekeeping gene, RPS9, and data were analyzed as delta cycle threshold (ΔCt). Primers are compiled in supplemental table 1.

Blood collected in heparin tubes and BAL cells were used for flow cytometry analysis using methods and antibodies previously described Hong et al.(2024).

### Statistics

Data were analyzed using the Mixed procedure of SAS 9.4 (SAS Institute, Cary, NC). The model included fixed effects of treatment and group. Contrast treatments were utilized to determine the effects of “Zn vs. No Zn,” “VA vs. No VA,” and “Intranasal (IN) treatment vs. CON.” For liver mineral and vitamin A, the pre challenge concentration served as a covariate in the analysis of the post challenge sample. Repeated measures were utilized to analyze plasma trace mineral and vitamin A concentrations and rectal temperatures; day was the repeated effect. Covariance structures were determined using the lowest AICC, compound symmetry was utilized. The Glimmix procedure of SAS 9.4 was utilized to analyze clinical and thoracic ultrasound score as repeated measures, day was the repeated effect. The covariance structures were unstructured and variance components for clinical and thoracic ultrasound scores, respectively. For viral shedding detection, data were analyzed as binary data using the Glimmix procedure with the fixed effects of treatment and group. Outliers were removed if greater than three standard deviations from the treatment means. Data were tested for normality and were log transformed for analysis if data were not normal (CellROX flow cytometry). Statistical significance is defined as *P* ≤ 0.05 and a tendency 0.1 ≥ *P* > 0.05.

## RESULTS

### Growth and Clinical Signs of Illness

**Figure 1.**
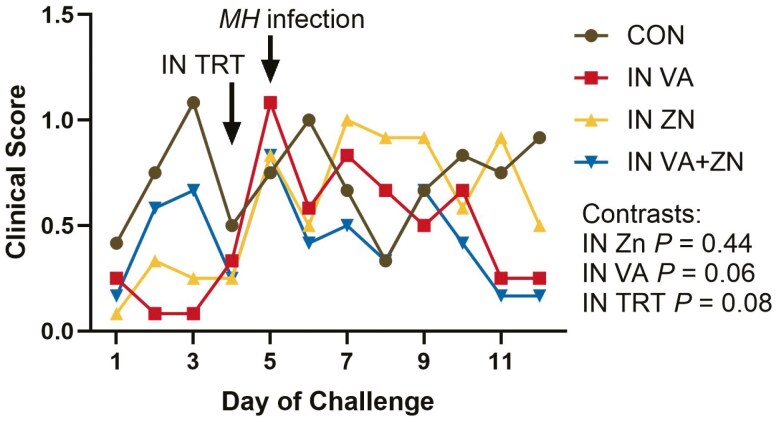
Effects of intranasal Zn and vitamin A treatments on clinical signs of infection. Clinical signs of infection scoring were performed daily by a trained observer blinded to treatment using methods described by McGuirk and Peek (2014). On d 0, all steers were challenged with bovine respiratory syncytial virus. On d 4, intranasal treatments (IN TRT) were administered including zinc (IN ZN; 50 mg Zn oxide nanoparticles], vitamin A (IN VA; 200,000 IU as retinyl palmitate nanoparticles), a combination of zinc (50 mg) and vitamin A (200,000 IU; IN VA + ZN) or no treatment (CON). On d 5, all steers were intratracheally inoculated with *Mannheimia haemolytica* (MH). Contrast statements were utilized to compare: CON and VA to ZN and VA + ZN (IN ZN), CON and ZN to VA and VA + ZN (IN VA), and CON to VA, ZN, and VA + ZN (IN TRT).

Treatment did not affect final body weight or average daily gain (ADG; *P *≥ 0.21). Disease symptoms were mild and total clinical scores tended to be decreased in VA treated calves (VA and VA + ZN; Figure 1; *P* = 0.06) and all treated calves compared to control (*P* = 0.08). There were no differences in rectal temperature (*P *≥ 0.30). Rectal temperature was greatest on d 0 (39.3 ± 0.05 °C) and lowest on d 7 (38.9 ± 0.05 °C), but the average rectal temperature was within normal ranges on all sampling days (day *P* < 0.01). Thoracic ultrasound scores were not affected by treatment (*P *≥ 0.27) but were affected by day (*P* = 0.01) where scores were increased on d 10 (0.4 ± 0.07) and 14 (0.3 ± 0.08) compared to d 0 (0.1 ± 0.07) and 4 (0.1 ± 0.07). On d 7, TUS scores were not different from all days (0.2 ± 0.07). There were no differences in the percent of steers shedding virus per treatment on all days (Supplemental table 2; *P *≥ 0.35).

### Vitamin A and Trace Mineral Concentrations

**Figure 2.**
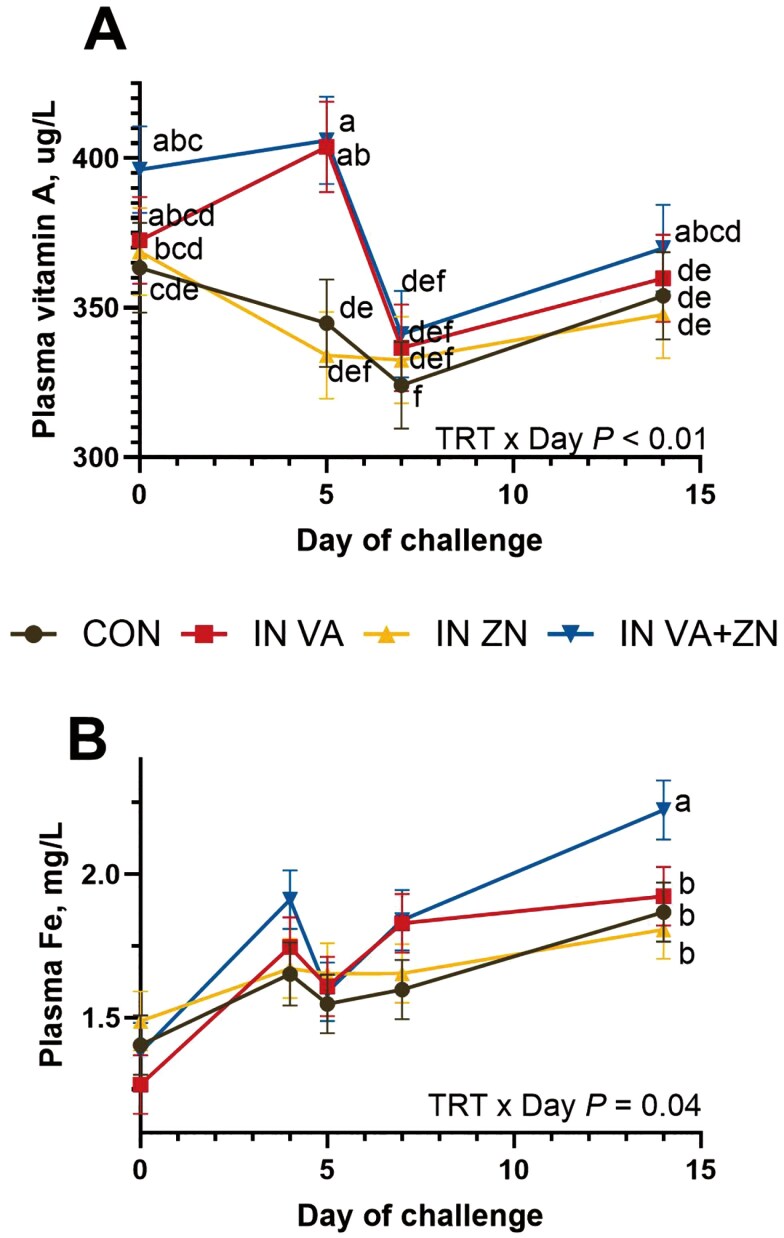
Effects of intranasal zinc and vitamin A treatments on plasma vitamin A and iron concentrations during respiratory disease challenge. On d 0, all steers were challenged with bovine respiratory syncytial virus. On d 4, intranasal treatments were administered including zinc (IN ZN; 50 mg Zn oxide nanoparticles], vitamin A (IN VA; 200,000 IU as retinyl palmitate nanoparticles), a combination of zinc (50 mg) and vitamin A (200,000 IU; IN VA + ZN) or no treatment (CON). On d 5, all steers were intratracheally inoculated with *Mannheimia haemolyti*ca. A. Plasma vitamin A concentrations were analyzed on d 0, 5, 7 and 14 of challenge. There was a TRT × Day interaction (*P *< 0.01) where VA treated steers (VA and VA + ZN) sustained plasma vitamin A concentrations on d 5, whereas non VA steers (ZN and CON) had decreased plasma vitamin A concentrations. ^abc^ Superscripts denote differences (*P* ≤ 0.05) in means across all treatments and days. B. Plasma Fe was analyzed on d 0, 4, 5, 7 and 14 post infection. There was a TRT × Day interaction (*P* = 0.04) where plasma Fe concentrations are similar for all treatments on d 0, 4, 5 and 7, but are increased in VA + ZN steers on d 14 compared to all other treatments. ^ab^ Superscripts denote differences (*P* ≤ 0.05) in means across treatments within day.

**Figure 3.**
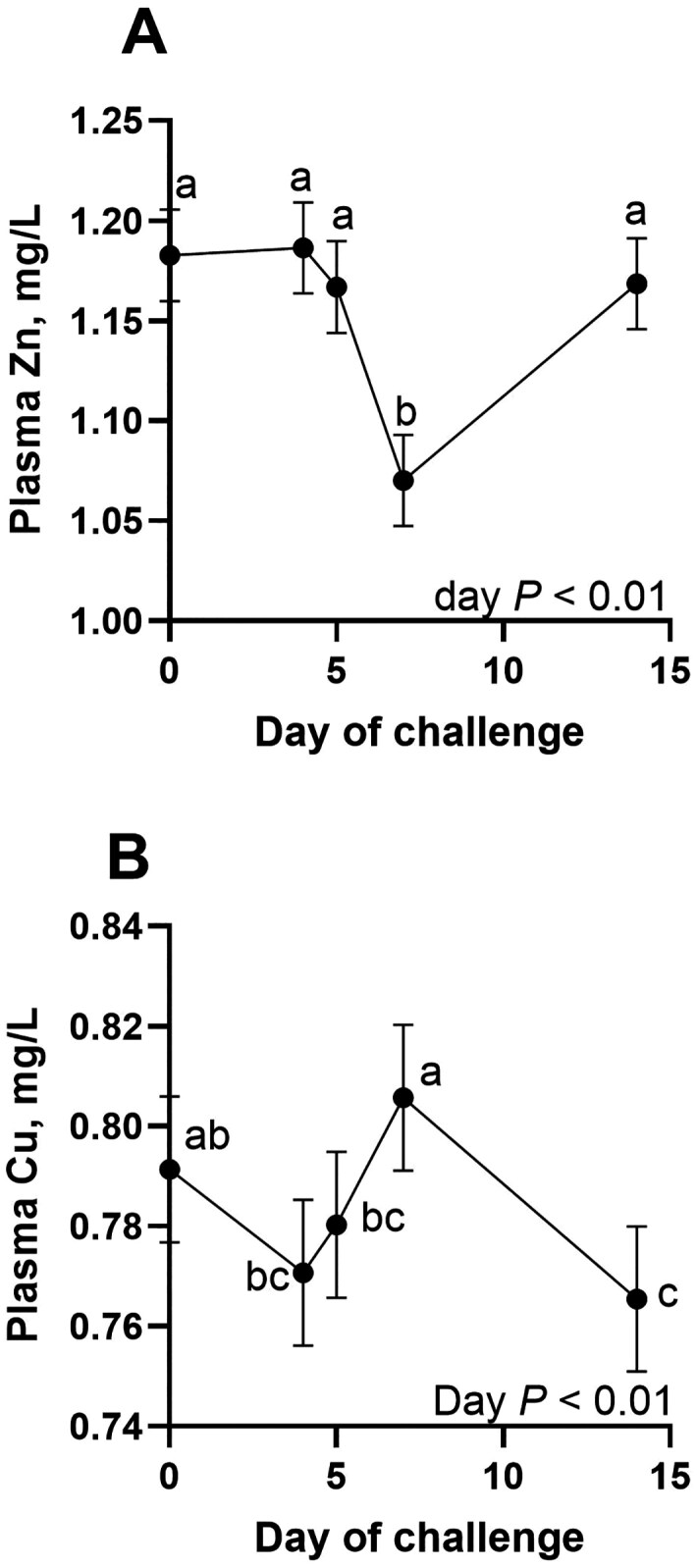
Effects of respiratory disease challenge on plasma zinc and copper concentrations. On d 0, all steers were challenged with bovine respiratory syncytial virus. On d 4, intranasal treatments were administered including zinc (IN ZN; 50 mg Zn oxide nanoparticles], vitamin A (IN VA; 200,000 IU as retinyl palmitate nanoparticles), a combination of zinc (50 mg) and vitamin A (200,000 IU; IN VA + ZN) or no treatment (CON). On d 5, all steers were intratracheally inoculated with *Mannheimia haemolyti*ca. Plasma Zn and Cu were analyzed on d 0, 4, 5, 7 and 14 of challenge. ^abc^ Superscripts denote difference (*P* ≤ 0.05) in means across days. A. There was an effect of day (*P* < 0.01) for plasma Zn where concentrations are lowest on d 7. B. There was an effect of day (*P* < 0.01) where plasma Cu slightly decreases on d 4, then increases to the highest concentration on d 7 followed by decreasing to the lowest concentration on d 14.

There was a TRT × Day effect (Figure 2; *P* < 0.01) where IN VA and IN VA + ZN steers maintained initial plasma vitamin A concentrations on d 5, while CON and IN ZN steers had decreased plasma vitamin A concentrations, however all steers had decreased plasma vitamin A on d 7, and increased to baseline on d 14. There was a TRT × Day effect for plasma iron (Fe; Figure 2; *P* = 0.04), where IN ZN + VA increased on d 4 and 14, and all other treatments had relatively similar plasma Fe concentrations, increasing throughout the study. There were no TRT × Day effects or effects of treatment on plasma copper (Cu; *P* ≥ 0.48) and Zn (*P* ≥ 0.89). Plasma Zn was lowest on d 7 (Figure 3; *P* < 0.01). Plasma Cu increased from d 5 to 7, then decreased on d 14 (Figure 3; *P* < 0.01).

Liver concentrations of Zn, manganese (Mn), Cu and Fe were not different before or after challenge (Table 3; *P* ≥ 0.12). Prior to infection, there were no differences in liver vitamin A (*P* ≥ 0.13). After challenge, there was an effect of Zn (*P* = 0.05) and vitamin A (*P* = 0.03) where steers treated with Zn tended to have lower liver vitamin A and steers treated with vitamin A tended to have increased liver vitamin A concentrations.

**Table 3.** Effects of intranasal zinc and vitamin A treatments on the liver trace mineral and vitamin A concentrations on a dry matter basis before and after bovine respiratory disease challenge

	Treatments^1^					Contrasts^2^		
Before challenge^3^	CON	IN VA	IN ZN	IN VA + ZN	SEM	ZN	VA	IN TRT
vitamin A, ug/g	32.4	29.5	30.4	33.8	3.65	0.76	0.94	0.78
Zinc, mg/kg	103	105	109	102	5.5	0.81	0.67	0.68
Mn, mg/kg	12.0	12.1	11.4	11.9	0.62	0.52	0.56	0.81
Cu, mg/kg	256	278	292	312	24.9	0.15	0.38	0.17
Fe, mg/kg	167	172	148	162	13.0	0.25	0.45	0.66
After challenge^4^								
vitamin A, ug/g	32.8	43.3	27.5	33.5	3.93	0.05	0.03	0.65
Zn, mg/kg	106	110	110	117	4.4	0.24	0.19	0.20
Mn, mg/kg	11.2	11.4	11.9	12.4	0.53	0.12	0.50	0.24
Cu, mg/kg	350	335	317	351	15.9	0.59	0.54	0.39
Fe, mg/kg	228	191	196	185	25.4	0.43	0.30	0.16

^1^Steers were inoculated with bovine respiratory syncytial virus on d 0. All intranasal treatments administered on d 4. CON = no intranasal treatment; IN VA = 200,000 IU as retinyl palmitate nanoparticles; IN ZN = 50 mg Zn oxide nanoparticles; IN VA + ZN = 50 mg Zn oxide and 200,000 IU of retinyl palmitate nanoparticles. Steers were intratracheally inoculated with *Mannheimia haemolytica* on d 5.

^2^Contrast statements were utilized to compare: CON and VA to ZN and VA + ZN (ZN), CON and ZN to VA and VA + ZN (VA), and CON to VA, ZN, and VA + ZN (IN TRT).

^3^Before challenge liver biopsy sample collected d −8 and −10 of viral challenge.

^4^After challenge liver biopsy sample collected on d 19 and 20 of viral challenge.

### Flow Cytometry

Cells from blood and BAL were isolated and stained for flow cytometry for cell populations and cell response to oxidative stress. On d 0, there was a tendency for increased CD11b on neutrophils (CH138+) in blood in steers that would be given intranasal treatment (Table 4; IN TRT *P* = 0.08), but no other differences in surface expression on neutrophils and CD14 cells (*P* ≥ 0.18). On d 7, there were no differences in CD14 and neutrophil populations in blood (Suppl. Table 3; *P* ≥ 0.18). On d 14, there was a tendency for increased CD11b expression on neutrophils in blood of Zn treated (Zn *P* = 0.08) and all treated steers (IN TRT *P* = 0.07), but there were no other differences in CD14 and CH138 (neutrophil) populations (*P* ≥ 0.19). In BAL cells on d 0, there was a tendency for steers who would receive VA (Table 5; VA *P* = 0.09) and any intranasal treatment (IN TRT *P *= 0.09) to have increased CD11b expression on CD14 cells. There was also a tendency for decreased expression of CD62L on CD14 in steers that would be given intranasal treatment in BAL cells (IN TRT *P* = 0.07). In the neutrophil population, there tended to be decreased CD11c expression in BAL from steers that would be treated with Zn (ZN *P* = 0.08), and all steers that would be given intranasal treatment (IN TRT *P* = 0.08). There were no other differences in the CD14 or neutrophil populations in BAL on d 0 (*P* ≥ 0.24). On d 7 there was decreased CD11c expression on CD14 cells (ZN *P* = 0.05). On d 7, there was increased CD11b expression on neutrophils in BAL cells of VA treated steers compared to steers not treated with VA (VA *P* = 0.01). On d 14, there tended to be increased CD11b expression on neutrophils in the Zn treated steers (ZN *P* = 0.02). In BAL neutrophils of all treated steers, CD11c was decreased on d 7 (IN TRT *P* = 0.04) and tended to be decreased on d 14 (IN TRT *P *= 0.06). There were no other differences in BAL CD14 populations on d 7 and 14 (*P* ≥ 0.14).

**Table 4.** Effects of intranasal zinc and vitamin A treatment on immune cell phenotype in blood

	Treatments^1^					Contrasts^2^		
	CON	IN VA	IN ZN	IN VA + ZN	SEM	ZN	VA	IN TRT
d0								
CD14, %	4.8	4.3	4.5	3.9	0.52	0.51	0.29	0.38
CD11b^3^	461	485	449	464	53.5	0.74	0.7	0.73
CD62L^3^	5562	5539	5521	5625	290.1	0.67	0.83	0.84
CD11c^3^	2265	2178	2133	2335	244.9	0.96	0.81	0.86
d7								
CD14, %	1.6	1.9	1.3	1.6	0.25	0.26	0.29	0.92
CD11b^3^	459	548	467	479	75.1	0.69	0.5	0.65
CD62L^3^	4917	4872	5090	5225	273.4	0.33	0.87	0.63
CD11c^3^	2058	2140	1776	1969	200	0.26	0.5	0.68
d14								
CD14, %	2.1	2.4	2.4	2.2	0.27	1	0.77	0.45
CD11b^3^	584	638	664	598	44.3	0.63	0.89	0.31
CD62L^3^	4549	4596	4848	4823	206.5	0.19	0.96	0.37
CD11c^3^	2820	2927	2717	2749	245	0.56	0.77	0.94
d0								
CH138, %	21.7	2.6	23.4	24.7	3.32	0.57	0.73	0.62
CD11b^3^	472	533	577	525	37.2	0.18	0.9	0.08
CD62L^3^	4168	4160	4296	4108	386.4	0.92	0.79	0.96
CD11c^3^	327	311	327	344	36.1	0.63	1	1
d7								
CH138, %	27.3	35.3	34	29.7	3.64	0.88	0.62	0.18
CD11b^3^	612	677	564	675	101.7	0.81	0.39	0.82
CD62L^3^	5191	4932	5374	4962	264.6	0.69	0.21	0.74
CD11c^3^	270	261	250	250	20.4	0.44	0.83	0.5
d14								
CH138, %	25.1	26.3	25.5	23.1	3.42	0.68	0.86	0.97
CD11b^3^	594	635	665	673	31.4	0.08	0.42	0.07
CD62L^3^	5102	5036	4765	4889	354.2	0.48	0.93	0.6
CD11c^3^	304	301	313	298	18.3	0.86	0.62	1

^1^Steers were inoculated with bovine respiratory syncytial virus on d 0. All intranasal treatments administered on d 4. CON = no intranasal treatment; IN VA = 200,000 IU as retinyl palmitate nanoparticles; IN ZN = 50 mg Zn oxide nanoparticles; IN VA + ZN = 50 mg Zn oxide and 200,000 IU of retinyl palmitate nanoparticles. Steers were intratracheally inoculated with *Mannheimia haemolytica* on d 5.

^2^Contrast statements were utilized to compare: CON and VA to ZN and VA + ZN (ZN), CON and ZN to VA and VA + ZN (VA), and CON to VA, ZN, and VA + ZN (IN TRT).

^3^Mean fluorescence index (MFI) of marker (CD11b, CD62L, or CD11c) within CD14 or CH138 populations.

**Table 5.** Effects of intranasal zinc and vitamin A treatment on the immune cell phenotype from bronchoalveolar lavage

	Treatments^1^					Contrasts^2^		
	CON	IN VA	IN ZN	IN VA + ZN	SEM	Zn	VA	IN TRT
d0								
CD14, %	3.1	3.1	3	3.5	0.61	0.73	0.68	0.84
CD11b^3^	616	745	683	729	54.7	0.62	0.09	0.09
CD62L^3^	1011	838	889	930	60.6	0.80	0.27	0.07
CD11c^3^	3780	3423	3257	3216	366.3	0.31	0.58	0.24
d7								
CD14, %	3.6	3.6	2.9	3.9	0.68	0.75	0.43	0.91
CD11b^3^	579	632	504	550	86.7	0.32	0.53	0.84
CD62L^3^	943	854	924	803	93.3	0.69	0.24	0.37
CD11c^3^	2742	2854	2704	2519	342.2	0.05	0.9	0.88
d14								
CD14, %	4.65	3.83	3.11	4.92	0.71	0.74	0.47	0.38
CD11b^3^	639	665	743	613	56.8	0.64	0.35	0.59
CD62L^3^	874	740	821	793	96.8	1	0.38	0.39
CD11c^3^	2666	3077	2495	2788	306.5	0.44	0.24	0.72
d0								
CH138, %	15.2	13.3	16.4	14.7	3.53	0.75	0.55	0.84
CD11b^3^	1085	1111	1032	1142	59	0.85	0.24	0.88
CD62L^3^	653	658	659	725	40.1	0.35	0.36	0.53
CD11c^3^	335	303	268	271	28.5	0.08	0.61	0.09
d7								
CH138, %	16.1	17.1	15	13.1	4.45	0.56	0.91	0.83
CD11b^3^	998	1143	678	1120	134.1	0.35	0.01	0.91
CD62L^3^	827	704	679	795	63.1	0.64	0.95	0.14
CD11c^3^	394	294	230	363	43.2	0.26	0.69	0.04
d14								
CH138, %	11.7	16.2	14.1	16.5	3.86	0.73	0.38	0.39
CD11b^3^	1222	1299	1510	1561	113	0.02	0.58	0.08
CD62L^3^	748	763	722	788	58.7	0.99	0.49	0.89
CD11c^3^	582	450	471	455	55.8	0.35	0.19	0.06

^1^Steers were inoculated with bovine respiratory syncytial virus on d 0. All intranasal treatments administered on d 4. CON = no intranasal treatment; IN VA = 200,000 IU as retinyl palmitate nanoparticles; IN ZN = 50 mg Zn oxide nanoparticles; IN VA + ZN = 50 mg Zn oxide and 200,000 IU of retinyl palmitate nanoparticles. Steers were intratracheally inoculated with *Mannheimia haemolytica* on d 5.

^2^Contrast statements were utilized to compare: CON and VA to ZN and VA + ZN (ZN), CON and ZN to VA and VA + ZN (VA), and CON to VA, ZN, and VA + ZN (IN TRT).

^3^Mean fluorescence index (MFI) of marker (CD11b, CD62L, or CD11c) within CD14 or CH138 populations.

On d 0, there were no differences due to treatment in the percent of live cells for cluster of differentiation (CD) 4+, CD8+, CD21 + and natural killer (NK) cells in circulation (Table 6; *P* ≥ 0.17). On d 7 post infection, NK cells were increased in circulation in CON compared to all treated steers (IN TRT *P* = 0.003) and lymphocytes tended to be decreased in all IN treated steers (IN TRT *P* = 0.07). There were no differences due to treatment for CD4+, CD8 + and CD21 + cells in circulation on d 7 (*P* ≥ 0.11). On d 14, Zn treated steers had increased CD21 + cells in circulation (ZN *P* = 0.04). Lymphocytes in circulation tended to be increased in Zn treated steers (ZN *P* = 0.08). There were no differences in CD4+, CD8 + and NK cells in circulation on d 14 (*P* ≥ 0.12).

**Table 6.** Effects of intranasal zinc and vitamin A treatments on lymphocyte populations in blood

	Treatments^1^					Contrasts^2^		
% of live	CON	IN VA	IN ZN	IN VA + ZN	SEM	ZN	VA	IN TRT
d0								
lymphocytes	61.2	63.7	63.3	59.3	3.51	0.75	0.83	0.81
CD4	11.3	11.3	11.7	10.2	0.89	0.66	0.40	0.80
CD8	10.9	7.6	8.0	10.3	1.45	0.96	0.72	0.17
CD21	8.2	7.3	7.4	8.1	1.54	1.00	0.96	0.73
NK	1.8	1.7	1.7	1.8	0.29	0.98	0.95	0.81
d7								
lymphocytes	57.9	48.8	53.0	53.0	3.04	0.92	0.13	0.07
CD4	10.1	8.8	8.6	9.0	0.68	0.34	0.52	0.11
CD8	4.9	4.4	2.8	4.7	0.66	0.20	0.27	0.23
CD21	6.7	4.9	4.9	6.3	0.95	0.80	0.84	0.20
NK	2.1	1.3	1.2	1.6	0.22	0.11	0.32	0.003
d14								
lymphocytes	53.8	50.8	53.9	59.7	2.63	0.08	0.57	0.73
CD4	9.9	9.7	10.3	10.9	0.68	0.26	0.76	0.65
CD8	10.9	10.1	7.8	10.2	1.01	0.12	0.39	0.17
CD21	4.4	5.0	6.1	5.8	0.63	0.04	0.80	0.09
NK	0.9	1.0	1.0	1.1	0.14	0.48	0.53	0.57

^1^Steers were inoculated with bovine respiratory syncytial virus on d 0. All intranasal treatments administered on d 4. CON = no intranasal treatment; IN VA = 200,000 IU as retinyl palmitate nanoparticles; IN ZN = 50 mg Zn oxide nanoparticles; IN VA + ZN = 50 mg Zn oxide and 200,000 IU of retinyl palmitate nanoparticles. Steers were intratracheally inoculated with *Mannheimia haemolytica* on d 5.

^2^Contrast statements were utilized to compare: CON and VA to ZN and VA + ZN (ZN), CON and ZN to VA and VA + ZN (VA), and CON to VA, ZN, and VA + ZN (IN TRT).

In BAL cells on d 0, steers who would be treated with VA tended to have decreased CD21 + cells (Table 7; VA *P* = 0.08), but there were no other differences (*P* ≥ 0.11). On d 7, steers treated with Zn had decreased CD8 + cells in BAL (ZN *P* = 0.01) and steers who received treatment had decreased CD8 + cells compared to CON (IN TRT *P* = 0.03). There was also a tendency for decreased CD4 + cells in BAL of Zn treated steers compared to non-Zn treated steers (ZN *P* = 0.06). There were no differences in the populations of CD21 + and NK cells in BAL on d 7 and 14 (*P* ≥ 0.18). On d 14, CON had increased CD4 + cells (IN TRT *P* = 0.05) and tended to have decreased CD8 + cells (IN TRT *P* = 0.06) compared to treated steers.

**Table 7.** Effects of intranasal zinc and vitamin A treatments on lymphocyte populations in bronchoalveolar lavage

	Treatments					Contrasts^2^		
% of live	CON	IN VA	IN ZN	IN VA + ZN	SEM	ZN	VA	IN TRT
d0								
CD4	30.4	32.6	29.7	29.7	29.55	0.39	0.64	0.93
CD8	29.7	31.4	28.4	29.2	2.32	0.44	0.56	0.99
CD21	27.5	19.4	23.9	19.0	3.70	0.58	0.08	0.11
NK	0.3	0.2	0.3	0.4	0.65	0.39	0.69	0.99
d7								
CD4	43.8	40.5	33.0	34.6	4.38	0.06	0.85	0.12
CD8	39.2	35.6	27.1	32.0	3.05	0.01	0.83	0.03
CD21	20.2	24.4	23.0	19.0	4.54	0.78	0.98	0.69
NK	2.1	1.5	2.5	1.4	0.89	0.86	0.33	0.79
d14								
CD4	39.4	31.9	28.5	33.9	3.59	0.19	0.75	0.05
CD8	39.1	32.3	31.5	34.6	2.83	0.32	0.48	0.06
CD21	27.0	34.5	30.1	25.6	2.92	0.31	0.60	0.38
NK	0.2	0.1	0.2	0.2	0.04	0.88	0.27	0.18

^1^Steers were inoculated with bovine respiratory syncytial virus on d 0. All intranasal treatments administered on d 4. CON = no intranasal treatment; IN VA = 200,000 IU as retinyl palmitate nanoparticles; IN ZN = 50 mg Zn oxide nanoparticles; IN VA + ZN = 50 mg Zn oxide and 200,000 IU of retinyl palmitate nanoparticles. Steers were intratracheally inoculated with *Mannheimia haemolytica* on d 5.

^2^Contrast statements were utilized to compare: CON and VA to ZN and VA + ZN (IN ZN), CON and ZN to VA and VA + ZN (IN VA), and CON to VA, ZN, and VA + ZN (IN TRT).

On d 0, steers that would be given intranasal Zn tended to have increased mean fluorescence intensity (MFI) for cellROX stained neutrophils in BAL (Table 8; ZN *P* = 0.10). There were no other differences in MFI for BAL and blood cells stained for calprotectin or cellROX on d 0 (*P *≥ 0.25). On d 7 in BAL cells, steers treated with Zn had increased calprotectin MFI on CD14 + and decreased cellROX MFI on CD14 + and neutrophils (ZN *P* ≤ 0.05). There was no difference due to treatment in the calprotectin MFI on neutrophils on d 7 in BAL cells (*P* ≥ 0.15). On d 14, steers who received intranasal treatment tended to have greater calprotectin MFI on CD14 + cells in BAL (IN TRT *P* = 0.06). There were no other differences in MFI in BAL cells on d 14 (*P* ≥ 0.29). There was no difference in the cellROX and calprotectin MFI of cells from blood on d 7 and 14 (Supplemental Table 3; *P* ≥ 0.24).

**Table 8.** Effects of intranasal zinc and vitamin A treatments on reactive oxygen species response in bronchoalveolar lavage cells

	Treatments^1^					Contrasts^2^		
MFI∆	CON	IN VA	IN ZN	IN VA + ZN	SEM	ZN	VA	IN TRT
d0								
CD14 Calprotectin	−104	−484	−617	−353	201.1	0.82	0.63	0.70
CD14 cellROX	−1658	−1439	−1368	−1590	448.5	0.87	1.00	0.70
CH138 Calprotectin	−102	5	−72	−96	108.2	0.71	0.67	0.66
CH138 cellROX	−1230	−1679	−944	−1099	264.2	0.10	0.25	0.97
d7								
CD14 Calprotectin	−107	−260	163	82	153.2	0.05	0.44	0.55
CD14 cellROX	4416	6247	432	1373	2287.9	0.05	0.52	0.48
CH138 Calprotectin	1	233	361	207	171.1	0.31	0.81	0.15
CH138 cellROX	3938	5459	590	1477	1445.8	0.01	0.38	0.36
d14								
CD14 Calprotectin	−95	205	333	−30	126.1	0.43	0.80	0.06
CD14 cellROX	−128	4411	588	−254	1993.2	0.29	0.33	0.41
CH138 Calprotectin	165	419	573	−136	209.0	0.70	0.24	0.57
CH138 cellROX	−26	2687	625	158	1118.7	0.40	0.31	0.33

^1^Steers were inoculated with bovine respiratory syncytial virus on d 0. All intranasal treatments administered on d 4. CON = no intranasal treatment; IN VA = 200,000 IU as retinyl palmitate nanoparticles; IN ZN = 50 mg Zn oxide nanoparticles; IN VA + ZN = 50 mg Zn oxide and 200,000 IU of retinyl palmitate nanoparticles. Steers were intratracheally inoculated with *Mannheimia haemolytica* on d 5.

^2^Contrast statements were utilized to compare: CON and VA to ZN and VA + ZN (ZN), CON and ZN to VA and VA + ZN (VA), and CON to VA, ZN, and VA + ZN (IN TRT).

### Gene Expression

Gene expression was analyzed in cells collected from the nasopharynx on d 0, 5 and 7. On d 0 in cells collected from NP swabs, steers that would receive vitamin A treatment had decreased expression of *IL-10* and *IL-8* (Table 9; VA *P* ≤ 0.03). Steers that would receive Zn had increased expression of surfactant protein-D (*SP-D*; Zn *P* = 0.005) in NP swabs on d 0. There were no other differences due to treatment on d 0 (*P* ≥ 0.13). On d 5, steers receiving any intranasal treatment had decreased expression of SAM pointed domain ETS factor (*SPDEF*; IN TRT *P* = 0.05) and tended to have decreased expression of *SP-D* (IN TRT *P* = 0.08) in NP swabs. There were no other differences in gene expression in NP swabs on d 0 (*P* ≥ 0.13). On d 5, steers receiving Zn tended to have increased expression of matrix metalloprotease 9 (*MMP9*) in NP swabs (ZN *P* = 0.06). There were no other differences in gene expression in NP swabs on d 5 (*P* ≥ 0.13). On d 7, vitamin A treated steers had decreased expression of *SP-D* (VA *P* = 0.04) and tended to have decreased expression of *MMP2* (VA *P* = 0.08) in NP swabs. On d 7, steers treated with Zn tended to have increased expression of cellular retinol binding protein 1 (*CRBP1*; ZN *P* = 0.08) in NP swabs. There were no other differences in gene expression in NP swabs on d 7 (*P* ≥ 0.13).

**Table 9.** Effects of intranasal zinc and vitamin A treatments on gene expression (∆ CT) in nasopharyngeal swabs

	Treatments^1^						Contrasts^2^		
	CON	IN VA	IN ZN	IN VA + ZN	SEM	*P-*value GRP	ZN	VA	IN TRT
d0									
SP-D	6.55	6.90	5.52	5.46	0.45	0.62	0.01	0.73	0.22
MMP2	7.63	8.26	8.46	7.20	0.34	0.01	0.70	0.31	0.33
MMP9	6.18	6.76	6.52	6.30	0.53	0.41	0.91	0.72	0.53
CRBP1	6.76	7.69	7.10	7.18	0.45	0.07	0.83	0.23	0.24
SPDEF	7.41	7.65	7.45	7.60	0.29	0.05	0.99	0.47	0.60
RALDH2	11.48	12.89	12.38	11.63	0.88	0.89	0.82	0.68	0.36
IL6	7.09	7.64	6.84	7.88	0.56	0.001	0.99	0.13	0.54
IL8	20.27	21.98	20.24	21.02	0.58	0.11	0.36	0.02	0.18
IL10	4.59	5.49	4.45	5.98	0.60	0.01	0.75	0.03	0.25
d5									
SP-D	7.19	10.08	8.60	7.98	1.22	0.68	0.72	0.24	0.08
MMP2	1.56	5.22	8.38	9.49	4.62	0.51	0.16	0.53	0.13
MMP9	3.22	3.03	1.84	2.45	0.52	0.07	0.06	0.69	0.18
CRBP1	2.36	2.12	1.36	2.12	0.40	0.01	0.20	0.51	0.28
SPDEF	6.75	8.16	8.13	6.93	0.45	0.15	0.86	0.81	0.05
RALDH2	-	-	-	-	-	-	-	-	-
IL6	6.30	6.35	5.90	6.12	0.22	0.13	0.14	0.52	0.49
IL8	24.63	25.52	25.02	26.00	0.64	0.02	0.47	0.13	0.23
IL10	2.41	2.56	1.78	2.26	0.41	0.004	0.24	0.42	0.65
d7									
SP-D	5.07	5.97	5.16	5.89	0.40	0.24	0.99	0.04	0.18
MMP2	7.39	7.87	7.51	8.20	0.35	0.94	0.49	0.08	0.20
MMP9	5.87	5.82	5.16	6.04	0.39	0.37	0.52	0.27	0.65
CRBP1	8.26	8.01	7.18	7.57	0.43	0.0001	0.08	0.87	0.17
SPDEF	7.02	7.54	7.24	7.29	0.25	0.25	0.95	0.25	0.23
RALDH2	11.96	10.60	11.89	11.40	1.55	0.89	0.75	0.42	0.59
IL6	5.79	6.27	6.42	6.24	0.46	0.84	0.51	0.74	0.32
IL8	20.36	21.34	20.59	21.12	0.49	0.01	0.99	0.13	0.24
IL10	3.81	3.52	3.13	3.84	0.50	0.29	0.71	0.67	0.57

^1^Steers were inoculated with bovine respiratory syncytial virus on d 0. All intranasal treatments administered on d 4. CON = no intranasal treatment; IN VA = 200,000 IU as retinyl palmitate nanoparticles; IN ZN = 50 mg Zn oxide nanoparticles; IN VA + ZN = 50 mg Zn oxide and 200,000 IU of retinyl palmitate nanoparticles. Steers were intratracheally inoculated with *Mannheimia haemolytica* on d 5..

^2^Contrast statements were utilized to compare: CON and VA to ZN and VA + ZN (ZN), CON and ZN to VA and VA + ZN (VA), and CON to VA, ZN, and VA + ZN (IN TRT).

CRBP1 = Cellular retinol binding protein 1; IL = interleukin; MMP = matrix metalloproteinase; RALDH2 = retinaldehyde dehydrogenase 2; SP-D = surfactant protein D; SPDEF = SAM pointed domain ETS factor.

Gene expression was analyzed in cells collected in BAL on d 0 and 7. Retinaldehyde dehydrogenase (*RALDH*) expression was increased in steers that would receive intranasal Zn on d 0 (Table 10; Zn *P* = 0.04). There were no other differences due to treatment on d 0 or 7 in the gene expression analyzed in BAL cells (*P* ≥ 0.11).

**Table 10.** Effects of intranasal zinc and vitamin A treatments on gene expression (∆ CT) in bronchoalveolar lavage cells

	Treatments^1^						Contrasts^2^		
	CON	IN VA	IN ZN	IN VA + ZN	SEM	*P-*value GRP	ZN	VA	IN TRT
d0									
SP-D	11.24	10.50	10.34	11.07	0.528	0.46	0.74	0.99	0.30
MMP2	11.86	11.71	11.18	11.81	0.624	0.05	0.65	0.71	0.69
MMP9	6.92	6.65	6.05	7.65	0.438	0.62	0.87	0.12	0.78
CRBP1	8.01	7.31	6.63	8.72	0.678	0.49	0.98	0.29	0.54
SPDEF	14.69	15.08	15.19	14.78	0.960	0.92	0.91	0.99	0.75
RALDH2	15.37	15.06	14.24	14.35	0.454	0.38	0.04	0.83	0.11
IL6	9.45	9.29	9.39	9.06	0.354	0.20	0.67	0.46	0.59
IL8	1.45	1.14	1.03	1.00	0.407	0.32	0.48	0.67	0.37
IL10	3.26	3.20	3.24	3.14	0.317	0.49	0.89	0.79	0.85
d7									
SP-D	10.50	10.81	10.37	10.73	0.536	0.78	0.84	0.51	0.81
MMP2	10.68	10.78	11.04	10.97	0.654	0.62	0.66	0.98	0.73
MMP9	7.14	6.89	7.72	7.33	0.459	0.39	0.25	0.46	0.73
CRBP1	7.75	8.20	8.30	7.56	0.468	0.01	0.91	0.74	0.59
SPDEF	13.99	13.77	15.06	14.58	0.619	0.07	0.12	0.55	0.48
RALDH2	15.78	15.92	15.82	16.24	0.502	0.01	0.70	0.55	0.69
IL6	9.77	9.36	9.66	10.28	0.457	0.69	0.36	0.81	0.99
IL8	1.39	0.68	1.28	1.62	0.525	0.46	0.41	0.72	0.74
IL10	2.86	2.85	3.10	3.13	0.185	0.82	0.16	0.96	0.42

^1^Steers were inoculated with bovine respiratory syncytial virus on d 0. All intranasal treatments administered on d 4. CON = no intranasal treatment; IN VA = 200,000 IU as retinyl palmitate nanoparticles; IN ZN = 50 mg Zn oxide nanoparticles; IN VA + ZN = 50 mg Zn oxide and 200,000 IU of retinyl palmitate nanoparticles. Steers were intratracheally inoculated with *Mannheimia haemolytica* on d 5.

^2^Contrast statements were utilized to compare: CON and VA to ZN and VA + ZN (ZN), CON and ZN to VA and VA + ZN (VA), and CON to VA, ZN, and VA + ZN (IN TRT).

CRBP1 = Cellular retinol binding protein 1; IL = interleukin; MMP = matrix metalloproteinase; RALDH2 = retinaldehyde dehydrogenase 2; SP-D = surfactant protein D; SPDEF = SAM pointed domain ETS factor.

## DISCUSSION

The current study utilized a challenge model modified from previous studies that have used BRSV (McGill et al., 2019; Mahmoud et al., 2020; Maina et al., 2023) and *MH* (Slate et al., 2021) independently and have resulted in BRD pathogenesis. In the current study, the BRD challenge resulted in subclinical clinical signs of BRD. Subclinical BRD is a major challenge for the beef industry, as these cattle may be more likely to hide their symptoms and treatment may be delayed or never received. Previous studies have reported nearly 70% of cattle that were never treated for respiratory disease in the feeding period had lung lesions at slaughter (Wittum et al., 1996). Animals with lung lesions at slaughter that had no visual signs of disease in the feedlot were shown to have decreased final body weight and ADG (Blakebrough-Hall et al., 2020). Due to decreased feedlot performance, subclinical BRD is a major economic burden for the beef industry (Griffin, 2014). Subclinical BRD symptoms observed in our study provide valuable insight into how Zn and vitamin A metabolism are affected by mild disease.

Vitamin A is associated with the development of healthy immune responses. In vitamin A deficiency, the immune response to vaccination and respiratory infection is dampened, indicated by lower concentrations of immunoglobulins (Rudraraju et al., 2014; Surman et al., 2014; McGill et al., 2019). Proinflammatory stimulus, such as an infection, can alter the circulating concentrations of retinol, with plasma retinol concentrations decreasing as acute phase protein concentrations in circulation increase (Thurnham et al., 2003). This has been observed in a study of BRSV infected calves with adequate vitamin A status at the time of infection where plasma retinol concentrations decreased after infection (McGill et al., 2019). In the current study, intranasal vitamin A as retinyl palmitate prevented the decline of plasma retinol concentrations until d 7. Vitamin A has previously been shown to be absorbed by non-gastrointestinal tissues when liver retinol concentrations increased after an intratracheal administration of vitamin A to piglets (Singh et al., 2010). Although sampling may not be timed to understand the absorption of intranasal vitamin A treatments, the current study also suggests absorption in the upper respiratory tract with vitamin A treated steers having greater liver retinol concentrations after disease challenge. Given these results, intranasal vitamin A could be of value in cattle with moderate or deficient vitamin A status since local administration seems to be absorbed and delays plasma concentrations from decreasing after viral inoculation.

Zinc has roles in immune cell development and function, epithelial barrier function and tissue repair. In humans, intranasal Zn treatments have been studied with varying outcomes, with some studies observing decreased severity and duration of symptoms (Hirt et al., 2000; Hunter et al., 2021) while others do not observe differences with intranasal Zn treatments (Belongia et al., 2001; Turner, 2001). In the current study, intranasal Zn did not affect disease outcomes, potentially because of the single dose of treatment, with many studies in humans having multiple doses of intranasal Zn treatment daily. For cattle, administering multiple doses of treatment may not be feasible due to the labor costs and potential stress of handling. One study investigated the use of intranasal Zn treatments as a mitigation strategy for BRD when administered upon arrival to the feedyard (Foster, 2016). They observed no differences in the frequency of BRD infection between Zn treated and control cattle. However, there were lesser interim ADG in cattle given Zn but no differences in final body weight and overall ADG were observed (Foster, 2016). The current study also did not observe differences in average daily gain or body weight across treatments.

In response to proinflammatory cytokines, Zn and Fe are sequestered into tissues, decreasing their concentrations in circulation and Cu concentrations in circulation will increase through mobilization from stores as part of the acute phase protein ceruloplasmin. In the current study, subclinical illness induced nutritional immunity. Plasma Fe was affected by day of challenge and treatment, with steers treated with ZN + VA and VA having the greatest percent change between timepoints (−12.3% from d 4 to 5). This is mediated through an upregulation of the iron regulatory hormone, hepcidin, by IL-6 (Nemeth et al., 2004). This occurs rapidly; therefore, it is not surprising that the timing of blood samples around the BRSV and *MH* inoculations do not capture the transient drop in plasma Fe concentrations, especially within subclinical disease conditions. Plasma Zn did not decrease in response to BRSV, but decreased on d 7 after the *MH* inoculation and returned to d 0 concentrations by d 14. Plasma Cu concentrations increased 4.5% from d 4 to 7, which could indicate an acute phase response (ceruloplasmin) after *MH* inoculation. Although there were no differences due to treatment for plasma Zn and Cu, subclinical disease progression and recovery did alter critical plasma trace mineral concentrations. Nutritional immunity has also been observed under more severe disease conditions where plasma Zn was decreased 7 d post *MH* inoculation and plasma Cu was increased on d 7 and 14 following *MH* inoculation (Wilson et al., 2016).

In the current study, gene expression of genes related to Zn, vitamin A and the immune response were evaluated in cells collected from BAL and NP swabs. Flow cytometry was also utilized to characterize the immune cell populations and oxidative response in blood and BAL cells. Although we did not expect differences on d 0, there were some differences in gene expression, immune cell populations and reactive oxygen species in response to stimulus, potentially due to transit induced stress. On d −1, steers were trucked for 6 hours to mimic arrival stress, and it is well understood that transit increases inflammation (Deters and Hansen, 2022). Increased inflammation would also induce a nutritional response to immune activation response, such as decreased plasma Fe concentrations, as observed in the current study on d 0, similar to that observed by Heiderscheit and Hansen (2022) the day after trucking. Future investigations may consider a non-challenge control to better understand the effects of pathogen inoculation and/or handling stress.

Intranasal Zn treatment influenced the expression of genes involved in Zn and vitamin A utilization. Gene expression of *matrix metalloproteinase 9* (*MMP9*) and *retinol binding protein 1* (*RPB1*) both require Zn for their downstream protein function (Mobarhan et al., 1992; Bode et al., 1993) and tended to be increased in NP cells of Zn-treated steers on d 5 and 7, respectively. Given RBP1 is critical for retinol transport (Blaner, 1989) and MMP9 plays a role in immune cell recruitment and lung tissue repair (Lemjabbar et al., 1999), these findings suggest Zn supplementation may support processes vital for vitamin A metabolism and tissue repair during a subclinical respiratory infection.

Surfactant protein-D (SP-D) can aid in pathogen clearance (Clark and Reid, 2002). Its gene expression has been upregulated in the presence of retinoic acid in vitro (Grubor et al., 2006). In the current study however, gene expression of *SP-D* in NP cells tended to be decreased in any steers with any intranasal treatment on d 5 and was decreased in steers treated with vitamin A on d 7. Lambs infected with MH tended to have decreased *SP-D* gene expression in lung homogenates (Grubor et al., 2004). It is possible that expected upregulation of *SP-D* by the presence of vitamin A is countered by subclinical disease conditions resulting in no change in expression due to vitamin A.

SAM pointed domain containing ETS transcription factor (*SPDEF*) regulates mucous production through signaling for goblet cell differentiation and can be upregulated by the proinflammatory cytokine IL-13 (Park et al., 2007). In the current study, *SPDEF* was upregulated in NP cells on d 5 in steers that did not receive intranasal treatment. This suggests intranasal Zn and vitamin A treatments may have modulated the inflammatory environment of the nasopharynx, leading to less expression of *SPDEF* in these steers on d 5.

Cells from blood and BAL were utilized for flow cytometry to determine the effects of intranasal treatments on cell populations. Neutrophils and macrophages are part of the innate branch of the immune system and are recruited to a site of infection to neutralize pathogens via reactive oxygen species production, phagocytosis, and forming extracellular traps. These cells can express proteins that increase their pathogen neutralization and recruitment to a site of infection, CD11b, CD11c and CD62L. In BAL cells on d 7, intranasal treatments increased the expression of CD11b on neutrophils (VA effect) and CD11c on macrophages (Zn effect) and neutrophils (all IN treatments effect). On d 14, there are more CD11b neutrophils in Zn treated steers and all treated steers tend to have lesser expression of CD11c on neutrophils. The altered expression of these molecules (CD11b, CD11c) in treated groups suggests intranasal treatments modulated the inflammatory environment within the lung, leading to changes in immune cell activation and recruitment. CellROX is a dye used in flow cytometry assays to assess the reactive oxygen species response of cells. Similarly, the reactive oxygen species response in BAL cells was affected by all intranasal treatment on d 7, where there was reduced CellROX from both neutrophil and macrophage populations, further suggesting an altered inflammatory environment due to intranasal Zn. On d 7, steers receiving intranasal Zn and all intranasal treatments had decreased frequency of CD8 + T cells and Zn treated steers tended to have decreased frequency of CD4 + T cells in BAL. Adaptive immune cells must be recruited to a site of infection, such as the lung, by proinflammatory cytokines. This further suggests intranasal treatments, specifically Zn, altered the inflammatory environment. In a more severe disease challenge, increasing the frequency of cells may aid in pathogen clearance, but during subclinical disease increased recruitment of cells may lead to additional tissue damage (Shi and Pamer, 2011).

Although there were no differences observed in growth performance across treatment, group 2 had lesser average daily gain compared to group 1. This difference, despite identical challenge protocols, may reflect the influence of environmental factors on the animals’ physiological and disease state. During the challenge, group 1 experienced lesser average temperatures (7.6 °C vs. 15.8 °C) and greater total rainfall (13.9 cm vs. 7.9 cm) compared to group 2. Weather conditions within the first week of arrival are recognized factors associated with BRD morbidity and mortality in feedlots (Terrell et al., 2011; Wisnieski et al., 2021). These environmental variations could similarly influence physiological responses, including baseline gene expression, which might then affect how animals respond to disease challenge or treatment. Although environmental conditions are unpredictable, it is interesting to consider the effects they can have on respiratory disease severity and outcomes and may need to be investigated further.

Under the conditions of this study, intranasal Zn and vitamin A treatments influenced lung inflammation and circulating micronutrient concentrations in Angus-cross steers experiencing subclinical BRD. Future research should evaluate intranasal treatments across diverse clinical conditions to fully assess their impact on BRD outcomes as ancillary therapies.

## Supplementary Material

txaf115_suppl_Supplementary_Materials_1
