# Supplementary material for: Intranasal zinc and vitamin A treatments alter response to bovine respiratory syncytial virus and Mannheimia haemolytica co-infection
Source: Transl Anim Sci. 2025 Aug 25;9:txaf115. doi: 10.1093/tas/txaf115 (PMC12416139; doi:10.1093/tas/txaf115)
Supplement: txaf115_suppl_Supplementary_Materials_1 [file txaf115_suppl_supplementary_materials_1.docx]

| **Supplementary table 1.** Primer sequences | | | | |
| --- | --- | --- | --- | --- |
| Gene^1^ | F | R | Accession no | citation |
| RPS9 | CGCCTCGACCAAGAGCTGAAG | CCTCCAGACCTCACGTTTGTTCC | NM_001101152.2 | (Sacco et al., 2012) |
| CRBP1 | CGGTCGACTTTACCGGGTACTG | GTCATGTCACTCATTCCTAGAGAC | NM_001025343 | (Lussier et al., 2017) |
| IL-10 | TTACCTGGAGGAGGTGATG | GTTCACGTGCTCCTTGATG | NM_174088.1 | (Sacco et al., 2012) |
| IL-6 | CTGAAGCAAAAGATCGCAGATCTA | CTCGTTTGAAGACTGCATCTC | NM_173921.2 | (Sacco et al., 2012) |
| IL-8 | TGTGAAGCTGCAGTTCTGTCAAG | TGCACCCACTTTTCCTTGGGGT | NM_173925.2 | (Talbott et al., 2014) |
| MMP2 | TTTGACATCATCTGC GGAGA | ACTCTCCAAGACCTTTTGCA |  | (Chowdhury et al., 2016) |
| MMP9 | GACCAGGACAAGCTCTACGG | CAGAAGCCCCACTTCTTGTC | NM_174744.2 | (Kliem et al., 2007) |
| RALDH2 | TCCCTGTCTGTAATCCAGCCAC | GAAAGCCAGCCTCCTTGATGAG |  | (Mohan et al., 2002) |
| SP-D | CCTGTACCCTGGTCATGTGT | AGCAGAGCCATTGTCTCCTT | NM_181026.2 | (Surlis et al., 2017) |
| SPDEF | CCCATCTGGACATCTGGAAATC | GAGGCGCAGAAGTGAATTGC | AY862877.1 | (Toffolatti et al., 2006) |
| ^1^ RPS9 = ribosomal protein S9; CRBP1 = cellular retinol binding protein; IL-10 = interleukin 10; IL-6 = interleukin 6; IL-8 = interleukin 8; MMP2 = matrix metalloproteinase 2; MMP9 = matrix metalloproteinase 9; RALDH2 = retinaldehyde dehydrogenase 2; SP-D = surfactant protein D; SPDEF = SAM pointed domain ETS factor | | | | |

| **Supplemental Table 2.** Effects of intranasal Zn and vitamin A treatments on bovine respiratory syncytial virus detected in nasal swabs as percent of each treatment group | | | | | | | | |
| --- | --- | --- | --- | --- | --- | --- | --- | --- |
|  | Treatments^1^ | | | |  | Contrasts^2^ | | |
| % with virus detected | CON | IN VA | IN ZN | IN VA + ZN | SEM | ZN | VA | IN TRT |
| Day of challenge^3^ |  |  |  |  |  |  |  |  |
| d 4 | 0.0 | 0.0 | 10.7 | 7.7 | 0.11 | 0.98 | 1.00 | 0.99 |
| d 5 | 24.5 | 16.2 | 24.5 | 41.4 | 14.41 | 0.35 | 0.85 | 0.91 |
| d 7 | 16.7 | 33.3 | 33.3 | 16.7 | 13.61 | 1.00 | 1.00 | 0.48 |
| d 10 | 0.0 | 0.0 | 8.3 | 8.3 | 7.98 | 0.98 | 1.00 | 0.99 |
| ^1^ All intranasal treatments administered on d 4. CON= no intranasal treatment; IN VA = 200,000 IU as retinyl palmitate nanoparticles; IN ZN = 50 mg Zn oxide nanoparticles; IN VA+ZN = 50 mg Zn oxide and 200,000 IU of retinyl palmitate nanoparticles. | | | | | | | | |
| ^2^ Contrast statements were utilized to compare: CON and VA to ZN and VA+ZN (IN ZN), CON and ZN to VA and VA+ZN (IN VA), and CON to VA, ZN, and VA+ZN (IN TRT) | | | | | | | | |
| ^3^Steers were inoculated with bovine respiratory syncytial virus on d 0. Intranasal treatments were administered on d 4. All steers were intratracheally inoculated with *Mannheimia haemolytica* on d 7. | | | | | | | | |

| **Supplemental Table 3.** Effects of intranasal zinc and vitamin A treatments on reactive oxygen species response in blood | | | | | | | | |
| --- | --- | --- | --- | --- | --- | --- | --- | --- |
|  | Treatments^1^ | | | |  | Contrasts^2^ | | |
| MFI | CON | IN VA | IN ZN | IN VA+ZN | SEM | ZN | VA | IN TRT |
| d0 |  |  |  |  |  |  |  |  |
| CD14 calprotectin | 194 | -12 | 24 | 252 | 149.3 | 0.75 | 0.94 | 0.53 |
| CD14 cellROX | 828 | 1076 | 839 | 690 | 667.8 | 0.78 | 0.94 | 0.96 |
| CH138 calprotectin | 208 | -726 | -333 | 353 | 411.5 | 0.50 | 0.76 | 0.34 |
| CH138 cellROX | 638 | 777 | 654 | 456 | 47.9 | 0.75 | 0.95 | 0.98 |
| d7 |  |  |  |  |  |  |  |  |
| CD14 calprotectin | 416 | 236 | 394 | 310 | 114.6 | 0.82 | 0.24 | 0.42 |
| CD14 cellROX | 961 | 940 | 1068 | 850 | 437.3 | 0.98 | 0.78 | 0.99 |
| CH138 calprotectin | 490 | 322 | 435 | 431 | 183.3 | 0.88 | 0.64 | 0.66 |
| CH138 cellROX | 589 | 585 | 652 | 469 | 282.8 | 0.93 | 0.74 | 0.95 |
| d14 |  |  |  |  |  |  |  |  |
| CD14 calprotectin | 81 | -90 | -56 | -210 | 257.3 | 0.62 | 0.53 | 0.51 |
| CD14 cellROX | -499 | -808 | -794 | -414 | 327.4 | 0.88 | 0.91 | 0.65 |
| CH138 calprotectin | 179 | 91 | 241 | 12 | 245.4 | 0.97 | 0.52 | 0.82 |
| CH138 cellROX | -183 | -333 | -174 | -192 | 214.5 | 0.72 | 0.69 | 0.84 |
| ^1^ Steers were inoculated with bovine respiratory syncyitial virus on d 0. All intranasal treatments administered on d 4. CON= no intranasal treatment; IN VA = 200,000 IU as retinyl palmitate nanoparticles; In ZN = 50 mg Zn oxide nanoparticles; IN VA+ZN = 50 mg Zn oxide and 200,000 IU of retinyl palmitate nanoparticles. Steers were intratracheally inoculated with *Mannheimia haemolytica* on d 5. | | | | | | | | |
| ^2^ Contrast statements were utilized to compare: CON and VA to ZN and VA+ZN ( ZN), CON and ZN to VA and VA+ZN (VA), and CON to VA, ZN, and VA+ZN (IN TRT) | | | | | | | | |
